# Supplementary material for: MetaRibo-Seq measures translation in microbiomes
Source: Nat Commun. 2020 Jun 29;11:3268. doi: 10.1038/s41467-020-17081-z (PMC7324362; doi:10.1038/s41467-020-17081-z)
Supplement: Supplementary file 10 — Supplementary Data 7 [file 41467_2020_17081_MOESM10_ESM.zip › File2/Confidence_High_Taxonomy/345712_out.krona.html]

Javascript must be enabled to view this page.

members
magnitude
magnitudeUnassigned
count
unassigned
taxon
rank

345712\_out

250

250
superkingdom
2

phylum
976
250

250
200643
class

order
171549
250

2005525
family
225

genus
5

SRS014415\_contig\_number\_625SRS064557\_contig\_number\_contig-100\_1202.122730SRS077641\_contig\_number\_16998SRS143070\_contig\_number\_16647SRS146813\_contig\_number\_8666
375288
225

203
species
46503

SRS011302\_contig\_number\_19138SRS011405\_contig\_number\_9228SRS013098\_contig\_number\_20201SRS013476\_contig\_number\_18351SRS013521\_contig\_number\_3140SRS013687\_contig\_number\_4125SRS013800\_contig\_number\_1953SRS013940\_contig\_number\_13833SRS013951\_contig\_number\_25067SRS014235\_contig\_number\_29077SRS014287\_contig\_number\_4251SRS014412\_contig\_number\_9126SRS014459\_contig\_number\_28751SRS014683\_contig\_number\_1155SRS015065\_contig\_number\_25756SRS015217\_contig\_number\_6764SRS015369\_contig\_number\_515SRS015579\_contig\_number\_17556SRS015694\_contig\_number\_6165SRS015782\_contig\_number\_14545SRS016056\_contig\_number\_1363SRS016203\_contig\_number\_443SRS016335\_contig\_number\_15337SRS016381\_contig\_number\_1358SRS016753\_contig\_number\_1645SRS016840\_contig\_number\_2183SRS017103\_contig\_number\_1809SRS017247\_contig\_number\_842SRS017433\_contig\_number\_10825SRS017521\_contig\_number\_37048SRS017701\_contig\_number\_1588SRS017821\_contig\_number\_19707SRS017859\_contig\_number\_561SRS018049\_contig\_number\_1285SRS018133\_contig\_number\_13268SRS018575\_contig\_number\_7046SRS018888\_contig\_number\_7272SRS018936\_contig\_number\_8584SRS019030\_contig\_number\_3680SRS019068\_contig\_number\_47586SRS019161\_contig\_number\_27656SRS019286\_contig\_number\_5169SRS019381\_contig\_number\_17012SRS019397\_contig\_number\_5819SRS019445\_contig\_number\_806SRS019685\_contig\_number\_37749SRS019808\_contig\_number\_contig-100\_482.112417SRS019968\_contig\_number\_16358SRS020233\_contig\_number\_42425SRS020271\_contig\_number\_1604SRS022524\_contig\_number\_9919SRS022609\_contig\_number\_contig-100\_1223.256919SRS023583\_contig\_number\_13834SRS024009\_contig\_number\_3548SRS024075\_contig\_number\_9829SRS024132\_contig\_number\_29012SRS024331\_contig\_number\_2598SRS024435\_contig\_number\_24203SRS024492\_contig\_number\_contig-100\_1050.105739SRS042966\_contig\_number\_15136SRS043667\_contig\_number\_1845SRS045004\_contig\_number\_12821SRS045244\_contig\_number\_2502SRS045645\_contig\_number\_25096SRS045713\_contig\_number\_4432SRS045739\_contig\_number\_4149SRS045826\_contig\_number\_13068SRS046068\_contig\_number\_1432SRS046369\_contig\_number\_10245SRS046717\_contig\_number\_2685SRS047014\_contig\_number\_21896SRS047044\_contig\_number\_12069SRS047433\_contig\_number\_11094SRS048164\_contig\_number\_15120SRS048981\_contig\_number\_2155SRS049402\_contig\_number\_9692SRS049446\_contig\_number\_3044SRS049712\_contig\_number\_17783SRS049896\_contig\_number\_8906SRS049959\_contig\_number\_37429SRS050026\_contig\_number\_956SRS050299\_contig\_number\_9425SRS050422\_contig\_number\_9634SRS050520\_contig\_number\_214SRS050752\_contig\_number\_12841SRS051031\_contig\_number\_25111SRS051610\_contig\_number\_9075SRS051882\_contig\_number\_12266SRS052078\_contig\_number\_1178SRS052697\_contig\_number\_8946SRS053356\_contig\_number\_28058SRS053649\_contig\_number\_1034SRS054059\_contig\_number\_4321SRS054352\_contig\_number\_13653SRS054905\_contig\_number\_1327SRS055966\_contig\_number\_343SRS056519\_contig\_number\_10290SRS057480\_contig\_number\_2091SRS058070\_contig\_number\_13150SRS062427\_contig\_number\_2781SRS062654\_contig\_number\_12191SRS063040\_contig\_number\_9291SRS063127\_contig\_number\_992SRS063190\_contig\_number\_308SRS063489\_contig\_number\_881SRS064232\_contig\_number\_370SRS064757\_contig\_number\_2041SRS065176\_contig\_number\_11696SRS065397\_contig\_number\_5275SRS065504\_contig\_number\_16674SRS074670\_contig\_number\_7547SRS075021\_contig\_number\_2158SRS075341\_contig\_number\_11696SRS075821\_contig\_number\_14307SRS076804\_contig\_number\_7342SRS076876\_contig\_number\_167SRS077024\_contig\_number\_3195SRS077086\_contig\_number\_6769SRS077231\_contig\_number\_23030SRS077552\_contig\_number\_4229SRS077589\_contig\_number\_5785SRS077849\_contig\_number\_16507SRS078176\_contig\_number\_20407SRS078242\_contig\_number\_contig-100\_742.183376SRS078665\_contig\_number\_9743SRS097920\_contig\_number\_23906SRS098073\_contig\_number\_698SRS098644\_contig\_number\_33351SRS098655\_contig\_number\_9058SRS098717\_contig\_number\_5349SRS101376\_contig\_number\_16323SRS103987\_contig\_number\_10429SRS104036\_contig\_number\_3728SRS1041039\_contig\_number\_10701SRS1041091\_contig\_number\_12227SRS1041092\_contig\_number\_9280SRS1041112\_contig\_number\_contig-100\_88.41760SRS1041116\_contig\_number\_1779SRS1041118\_contig\_number\_12327SRS1041130\_contig\_number\_1742SRS1041134\_contig\_number\_9513SRS1041136\_contig\_number\_10523SRS1041137\_contig\_number\_22994SRS1041138\_contig\_number\_2686SRS1041139\_contig\_number\_2791SRS1041140\_contig\_number\_20980SRS1041141\_contig\_number\_7461SRS104311\_contig\_number\_7833SRS104327\_contig\_number\_621SRS104400\_contig\_number\_34569SRS104912\_contig\_number\_20430SRS104975\_contig\_number\_12788SRS105082\_contig\_number\_11244SRS105153\_contig\_number\_15166SRS1054716\_contig\_number\_12381SRS1055022\_contig\_number\_21448SRS1055069\_contig\_number\_8250SRS1055095\_contig\_number\_10SRS140492\_contig\_number\_14547SRS140513\_contig\_number\_9531SRS142503\_contig\_number\_15896SRS142542\_contig\_number\_13672SRS142923\_contig\_number\_13906SRS142980\_contig\_number\_635SRS143148\_contig\_number\_25477SRS143181\_contig\_number\_149SRS143372\_contig\_number\_6301SRS143523\_contig\_number\_8675SRS143722\_contig\_number\_15393SRS143780\_contig\_number\_15418SRS143876\_contig\_number\_1686SRS143991\_contig\_number\_24671SRS144135\_contig\_number\_26118SRS144297\_contig\_number\_9453SRS144537\_contig\_number\_34149SRS144603\_contig\_number\_11363SRS144714\_contig\_number\_27762SRS145497\_contig\_number\_18710SRS146764\_contig\_number\_19049SRS146812\_contig\_number\_contig-100\_1455.306231SRS147346\_contig\_number\_9986SRS147425\_contig\_number\_3032SRS147557\_contig\_number\_29202SRS147614\_contig\_number\_7544SRS147977\_contig\_number\_670SRS148091\_contig\_number\_9756SRS148319\_contig\_number\_3919SRS148424\_contig\_number\_15966SRS148721\_contig\_number\_49949SRS148784\_contig\_number\_21256SRS148817\_contig\_number\_1423SRS149325\_contig\_number\_2647SRS893170\_contig\_number\_352SRS893230\_contig\_number\_6874SRS893252\_contig\_number\_12670SRS893292\_contig\_number\_1052SRS893295\_contig\_number\_4216SRS893300\_contig\_number\_2137SRS893327\_contig\_number\_1763SRS893358\_contig\_number\_4103SRS893378\_contig\_number\_10387SRS893382\_contig\_number\_1481SRS893383\_contig\_number\_33428

8
1750560
species

SRS022137\_contig\_number\_11123SRS049773\_contig\_number\_28245SRS050925\_contig\_number\_27837SRS055982\_contig\_number\_11229SRS143895\_contig\_number\_44020SRS147271\_contig\_number\_23610SRS148159\_contig\_number\_32998SRS148196\_contig\_number\_10116

8

SRS019910\_contig\_number\_17480SRS043841\_contig\_number\_811SRS044535\_contig\_number\_12593SRS048870\_contig\_number\_contig-100\_460.128337SRS052576\_contig\_number\_contig-100\_751.752SRS098571\_contig\_number\_31734SRS1055076\_contig\_number\_16052SRS145308\_contig\_number\_1198
1263094
species

1

SRS063985\_contig\_number\_15701
1897013
species

family
171552
1

genus
838
1

1

SRS049995\_contig\_number\_25136
species
165179

1
family
171550

239759
genus
1

1

SRS053398\_contig\_number\_12660
species
1262694

21
family
815

21
genus
816

3
species
821

SRS014948\_contig\_number\_17039SRS018836\_contig\_number\_17202SRS097889\_contig\_number\_17060

3
28116
species

SRS075984\_contig\_number\_3331SRS101433\_contig\_number\_3479SRS149075\_contig\_number\_17890

469585
species

SRS148253\_contig\_number\_2442
1

246787
species

SRS058770\_contig\_number\_1244
1

species
817

SRS078419\_contig\_number\_20909SRS144506\_contig\_number\_33695
2

1
species
47678

SRS023914\_contig\_number\_15698

1

SRS064276\_contig\_number\_16328
2292935
species


SRS015264\_contig\_number\_17942
1638780
species
1


SRS012969\_contig\_number\_4603SRS015663\_contig\_number\_7080SRS076976\_contig\_number\_5615SRS149181\_contig\_number\_941
species
46506
4

1

SRS016132\_contig\_number\_248
species
820

species
2292944

SRS050941\_contig\_number\_21229
1


SRS014613\_contig\_number\_11870SRS147139\_contig\_number\_31317
818
species
2

2
1853231
family

1
genus
574697

1
544644
species

SRS023526\_contig\_number\_31427

genus
283168
1


SRS104636\_contig\_number\_15302
species
626933
1
